# Correlation between Drop Impact Energy and Residual Compressive Strength According to the Lamination of CFRP with EVA Sheets

**DOI:** 10.3390/polym12010224

**Published:** 2020-01-16

**Authors:** Sun-Ho Go, Min-Sang Lee, Chang-Gi Hong, Lee-Ku Kwac, Hong-Gun Kim

**Affiliations:** 1 Department of Mechanical Engineering, Graduate School, Jeonju University, Jeonju-si 55069, Korea; exotherm@daum.net (S.-H.G.); lovely-lms@hanmail.net (M.-S.L.); 2 Department of Carbon Fusion Engineering, Graduate School, Jeonju University, Jeonju-si 55069, Korea; proud117@naver.com; 3 Department of Automotive Engineering, Jeonju University, Jeonju-si 55069, Korea

**Keywords:** carbon-fiber-reinforced plastic, building materials, impact, ethylene vinyl acetate

## Abstract

Carbon-fiber-reinforced plastic is an important building material; however, its application is limited because of its brittleness, leading to vulnerability under shock. Thus, the strength performance of carbon-fiber-reinforced plastics needs to be improved. Here, the drop impact test was conducted to analyze the impact energy and fracture characteristics of carbon-fiber-reinforced plastics and ethylene vinyl acetate sheets. The compression after impact test was performed to assess the residual compressive strength. The thermal energy generated was measured as change in temperature at the time of fracture to investigate the relationship between thermal and mechanical properties. The impact absorption efficiency of 100% was achieved when the carbon-fiber-reinforced plastics specimen was laminated with four or more sheets of ethylene vinyl acetate. The thermal energy generated during impact, the impact load, and the compression after impact test strength was reduced with the increasing number of laminated ethylene vinyl acetate layers. Our results showed that, by carefully selecting the optimal conditions of fabricating the carbon-fiber-reinforced plastic/ethylene vinyl acetate composites, carbon composite materials can be used for impact mitigation.

## 1. Introduction

Carbon-fiber-reinforced plastic (CFRP) is an important material for aircraft and aerospace industries because of its numerous advantages such as high specific strength, non-rigid specific stiffness, and excellent corrosion and chemical resistance. It also provides the strength and rigidity required for the design of structures according to the composite lamination method, fiber arrangement, and mixing ratio of the base material. However, CFRP is vulnerable to shock because of the brittleness and macroscopic interface of the carbon fiber and matrix. Thus, its performance against impact needs to be improved. The impact level of the composite depends on the speed of impact. Local impact damage occurs because of the lack of time for the composite to respond to the impact because the speed of impact increases from a low speed of 1–100 m/s to a higher speed. In the case of low-speed shock, micro-damage is often caused instead of visible damage. In addition, because CFRP has an excellent elastic force, it absorbs all kinetic energy of the impactor as absorbed energy under low-speed impact, and then transfers other energy back to the impactor through elastic restoration [1,2,3,4,5].

To improve the fracture resistance and material flexibility of CFRP-based composites, in combination with CFRP, ethylene vinyl acetate (EVA) sheets were laminated with a CFRP prepreg. EVA sheets include copolymers of ethylene and a vinyl acetate monomer, which has excellent flexibility. Furthermore, EVA exhibits excellent impact resistance and abrasion resistance because of the molecular ring of polyethylene [6,7].

In this study, a drop impact test was conducted to investigate the impact energy and fracture characteristics of brittle CFRP and ductile EVA sheets according to the lamination method. A compressive compression after impact test (CAI) was conducted after the impact to obtain the residual compressive strength. In each test, the thermal energy generated at the time of breakage was visualized using a thermal imaging camera to investigate the relationship between thermal and mechanical properties.

## 2. Materials and Methods

### 2.1. Specimen Preparation

**Figure 1:**
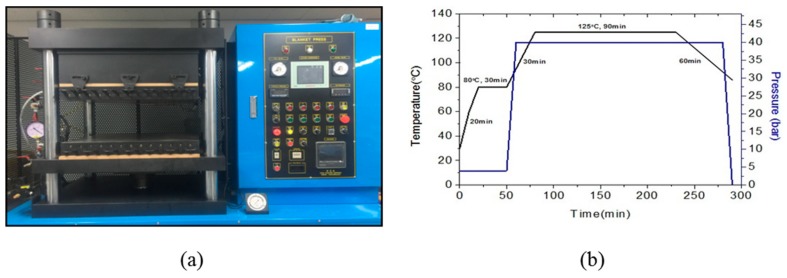
The set-up for specimen manufacturing: (**a**) autoclave machine, and (**b**) curing cycle of carbon-fiber-reinforced plastic (CFRP) stacking specimen.

The test specimen CFRP used in the study was carbon prepreg WSN-3k (SK Chemical, Seongnam, South Korea), and the chemical properties of the material are shown in Table 1. The mechanical properties of the flexible EVA sheet (Hwasung Industry, Seoul, South Korea) are shown in Table 2. In order to have similar thickness of the composites, three specimens were laminated and fabricated according to five types of lamination methods, as shown in Table 3. The laminated material was molded by the hot-press mold method, and the molding equipment and process conditions are shown in Figure 1a,b.

### 2.2. Drop Weight Impact and Compression after Impact Test

The fracture tendency by the drop impact of the polymer material was confirmed to show three modes of failure in a previous study [6,8]. These three modes include the puncture failure mode, crack failure mode, and brittle failure mode [6]. Usually, the three modes of damage may involve either internal damage or delamination from the interface or external damage or combination of the three damages. In the puncture failure mode (P-mode), the fracture completely penetrates to the extent that the diameter of the fracture site and the diameter of the weight are almost the same. This fracture behavior is observed in the case of polycarbonate and polyethylene. In the crack failure mode (C-mode), the crack radially propagates to the surrounding areas after the site hit by the weight is deformed. It is a form of fracture found in polypropylene and propylene–ethylene block copolymers. In the brittle fracture mode (B-mode), the crack propagates radially from the center of the specimen. Such fracture behavior is observed in polystyrene and is known to exhibit a small value of impact absorption energy [9,10,11].

The test conditions and specimen specifications were chosen in accordance with the American society for testing and materials (ASTM) D 7136 for the drop impact test. The results analysis was performed with three specimens per type.

In drop weight impact behavior analysis, the impact energy (*J_i_* (in Joule)) and the absorbed energy (*J_a_* (in joule)) can be calculated using Equations (1) and (2), respectively.

(1) $$J_{i}=\frac{m\;\times \;v_{i}{}^{2}}{2},$$

(2) $$J_{a}\left(t\right)=\frac{m\times \;\left(v_{i}{}^{2}-\;v{\left(t\right)}^{2}\right)}{2}+m\times g\times d\left(t\right),$$

where *m* is the impact weight (kg), *v_i_* is the initial impact speed (m/s), *v*(*t*) is the impact speed as a function of time, *g* is the acceleration due to gravity (9.87 m/s^2^), and *d*(*t*) is the impact displacement (m).

The impact absorption efficiency of the material was analyzed using impact energy and impact absorbed energy obtained through the drop impact test to compare the shock absorption performance under equivalent impact energy conditions. The impact absorption efficiency was calculated as follows:

(3) $$e_{abs}\left(\%\right)=\frac{J_{a}}{J_{i}}\times 100.$$

**Figure 2:**
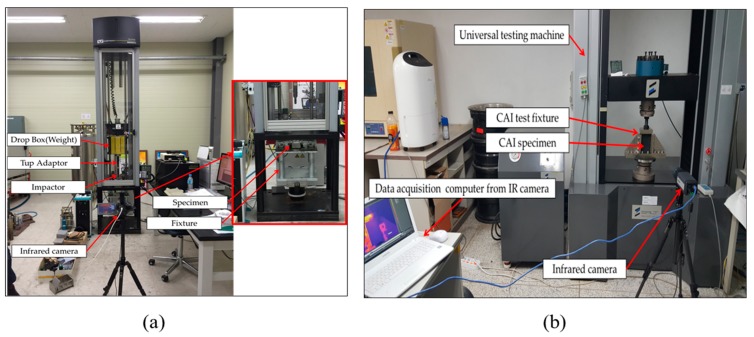
Set-up of experiments: (**a**) drop weight testing, and (**b**) compression after impact (CAI) testing.

At the same time, the relationship between impact energy and thermal energy was analyzed by converting the energy generated by the impact into heat using a thermal imaging camera. Moreover, because the flow of impact energy is visualized as temperature and is represented by a thermal image, the thermal image was used to investigate the fracture mode. The test conditions are summarized in Table 4, and the test equipment is shown in Figure 2a. The maximum temperature at the time of breakage was derived for a comparative analysis [8,12,13,14].

The compression after impact (CAI) test was performed according to ASTM D 7137. A CAI test is conducted to confirm the matrix cracking and fiber breakage for lateral shear and vertical stress after impact damage of laminated composite materials and to verify the strength against compressive load after impact [15,16]. In particular, interlayer separation is known to reduce the compressive strength of laminated composites by 40% to 60%. Test results were calculated using the compressive load and the cross-sectional area of the specimen with the CAI strength. The CAI test was conducted by installing a jig, as shown in Figure 2b. For the CAI test, the temperature distribution was monitored using a thermal imaging camera. For temperature results, the maximum temperature at the time of breakage at room temperature was derived for comparative analysis [15,16,17,18,19].

## 3. Results and Discussion

### 3.1. Drop Impact Test Results

**Figure 3:**
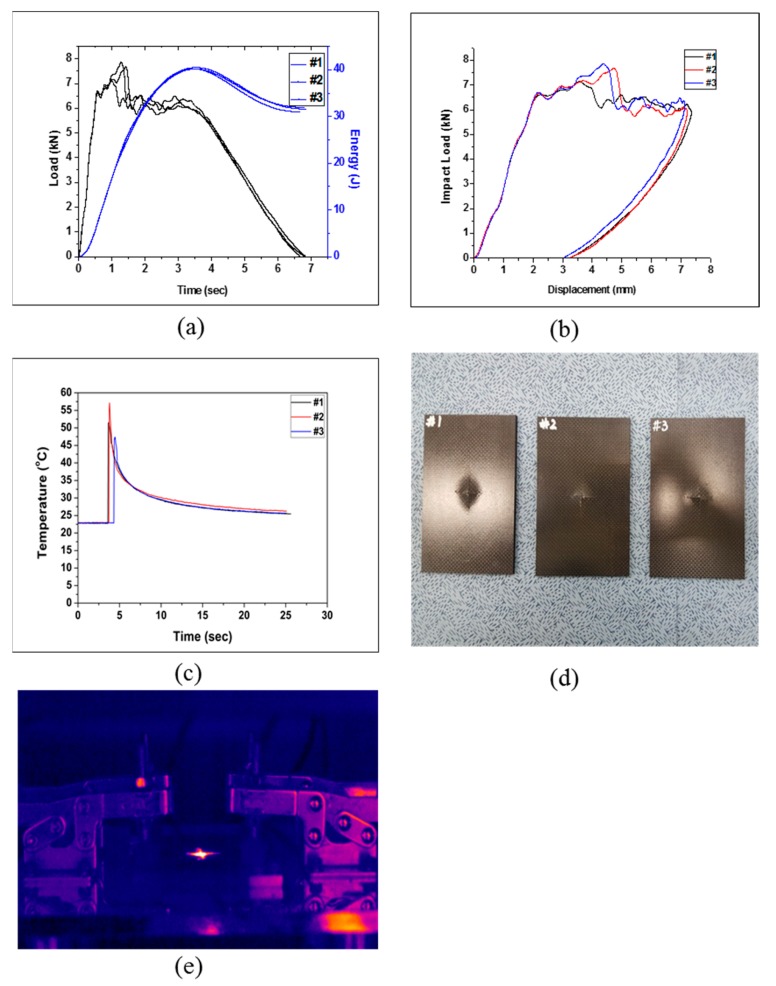
Impact test results of Type 1: (**a**) changes in load and energy with time, (**b**) impact load and displacement curve, (**c**) changes in temperature with time, (**d**) impact test of specimen Type 1, and (**e**) infrared (IR) image of the impact test (Type 1).

The test was conducted with three test specimens for each lamination type. The impact load, impact energy, impact absorption energy, impact absorption efficiency, and temperature were obtained for each specimen for a comparative analysis. Specimen Type 1 was prepared by laminating 24 plies of pure CFRP. The test results are shown in Figure 3 and Table 5. In the displacement–load curve in Figure 3b, the displacement was found to decrease after the maximum displacement. The displacement was probably restored because the energy other than the absorbed energy was transferred to the impactor by the elastic restoration of the specimen. The drop test results showed that the fracture formed crosswise from its beginning point, which was confirmed by observing the specimen after the drop test and its thermal image, as shown in Figure 3d,e. The temperature derived by the thermal imaging camera for each type was compared by deriving the difference between the initial temperature of the test and the energy radiated during the impact. An average impact energy of 40.28 J was applied, and the absorbed energy was 31.50 J, resulting in an energy absorption efficiency of 78.2%.

**Figure 4:**
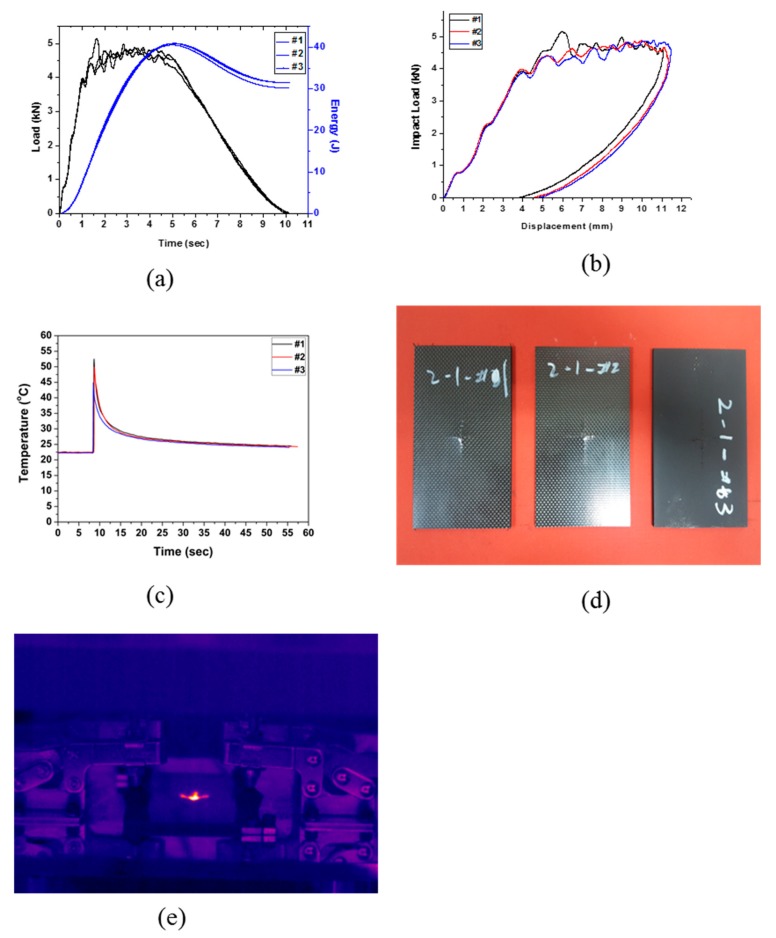
Impact test results of Type 2: (**a**) changes in load and energy with time, (**b**) impact load and displacement curve, (**c**) changes in temperature with time, (**d**) impact test of specimen Type 2, and (**e**) IR image of impact test (Type 2).

The specimen Type 2 was prepared by cross-laminating 20 plies of CFRP and two plies of EVA. The drop test results are shown in Figure 4 and Table 6. As for composite Type 1, in the displacement–load curve shown in Figure 4b, the displacement decreased after the maximum displacement was reached, suggesting that energy other than the absorbed energy was transferred back to the impactor. As can be seen in Figure 4d,e, the results of the drop test showed that the fracture formed crosswise from the center, similar to the fracture formation of Type 1. An average impact energy of 40.82 J was applied, and the impact absorption energy was 31.06 J, resulting in an energy absorption efficiency of 76.09%. The impact absorption performance was considered to be the same as that of Type 1. Therefore, it was deduced that the impact absorption efficiency cannot be improved with lamination of two plies of EVA sheet.

**Figure 5:**
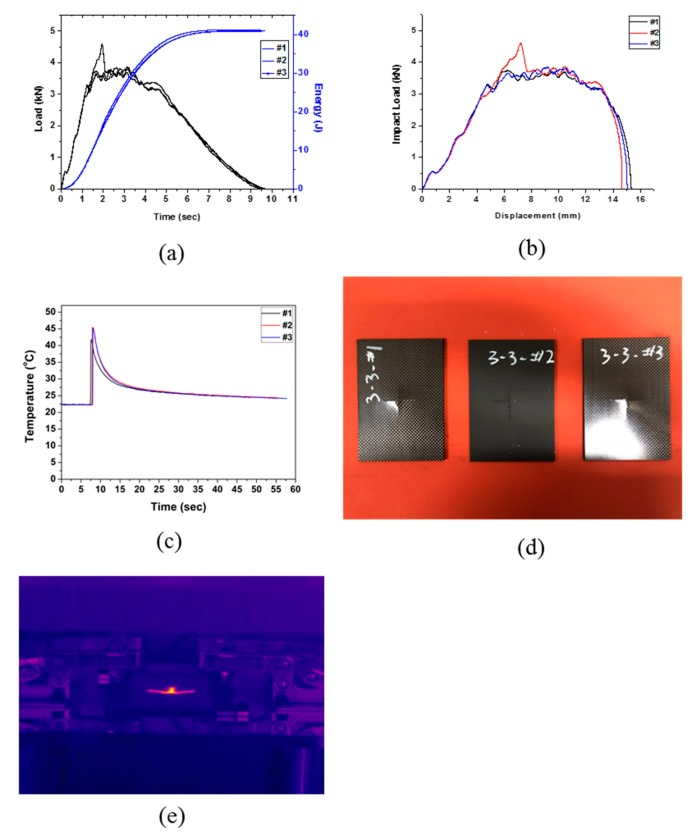
Impact test results of Type 3: (**a**) changes in load and energy with time, (**b**) impact load and displacement curve, (**c**) changes in temperature with time, (**d**) impact test of specimen Type 3, and (**e**) IR image of impact test (Type 3).

The specimen Type 3 was prepared by cross-laminating 16 plies of CFRP and four plies of EVA. The drop test results are shown in Figure 5 and Table 7. As seen in the load–displacement curve (Figure 5b), the impact energy was completely absorbed by the specimen as the displacement increased until the end of the test, unlike in the previous two types. Type 3 was also found to be fractured crosswise from the center (see Figure 5d,e). An average impact energy of 41.05 J was applied, all of which was absorbed, giving an impact absorption efficiency of 100%. It seems that the increase in number of flexible EVA sheets from two sheets to four sheets had a dominant effect on the enhancement of impact energy absorption.

**Figure 6:**
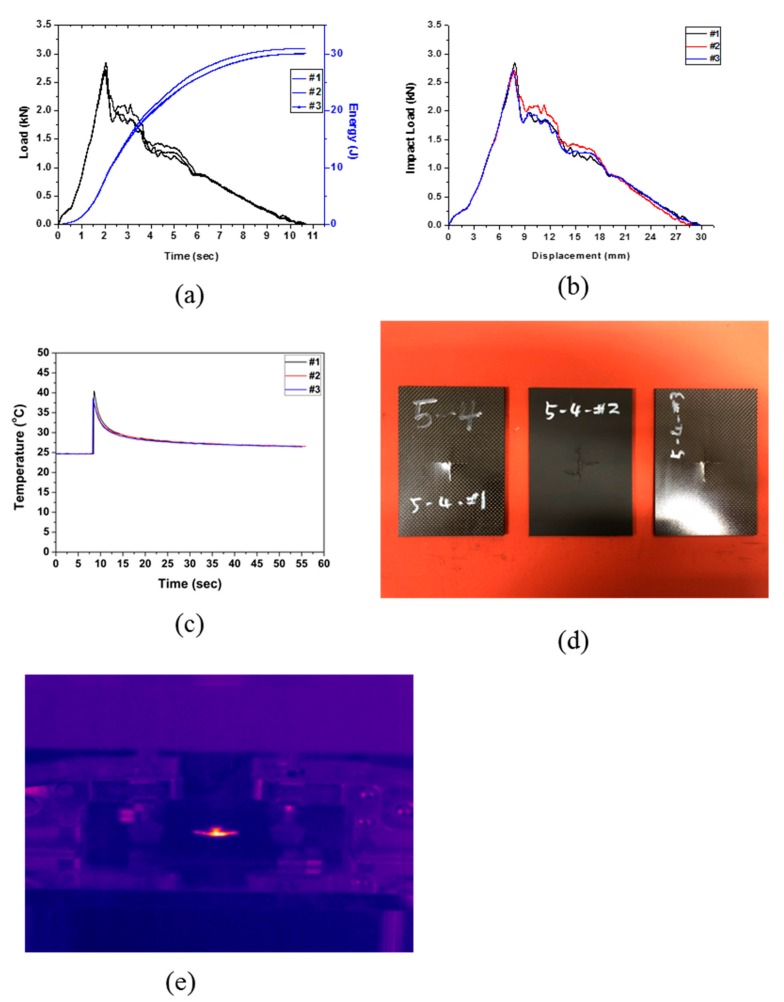
Impact test results of Type 4: (**a**) changes in load and energy with time, (**b**) impact load and displacement curve, (**c**) changes in temperature with time, (**d**) impact test of specimen Type 4, and (**e**) IR image of impact test (Type 4).

Specimen Type 4 was prepared by laminating eight plies of CFRP and eight plies of EVA at the center of the upper part and the lower part in symmetry from the center part. The drop test results are shown in Figure 6 and Table 8. As can be seen in the time–energy curve (see Figure 6a) and the load–displacement curve (Figure 6b), the impact energy was completely absorbed by the specimen. The same fractures behavior was observed as in Type 3 where the crack propagated crosswise from the center to both sides, as can be seen in Figure 6d,e. An average impact energy of 30.32 J was applied, all of which was absorbed, resulting in an impact absorption efficiency of 100%. In comparison with Type 3, Type 4 had lower average impact energy required to fracture to composite. This may be attributed to large number of EVA sheets in the composite.

**Figure 7:**
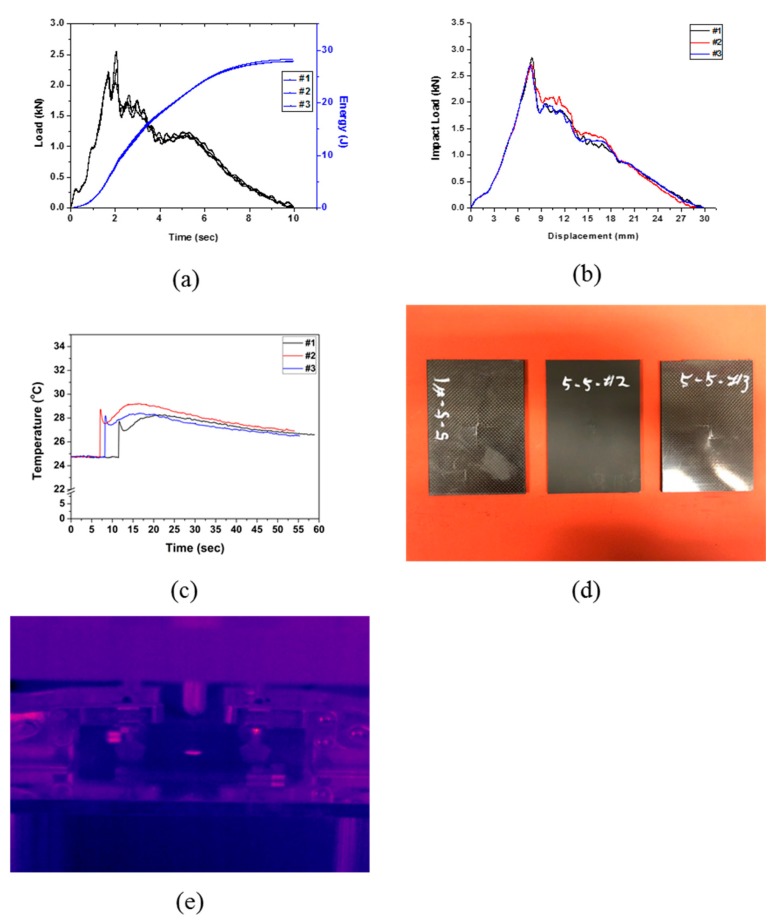
Impact test results of Type 5: (**a**) changes in load and energy with time, (**b**) impact load and displacement curve, (**c**) changes in temperature with time, (**d**) impact test of specimen Type 5, and (**e**) IR image of impact test (Type 5).

Specimen Type 5 had the same number of laminated layers as Type 4, but the EVA sheets were symmetrically laminated on the outside from the center. The drop test results of Type 5 are shown in Figure 7 and Table 9. An average impact energy of 28.00 J was applied, and the energy absorption was 28.00 J, resulting in an impact absorption efficiency of 100%. Such results can also be deduced from the time–energy curve (Figure 7a) and the load–displacement curve (Figure 7b), where it seems that the impact energy was completely absorbed by the specimen as observed for the Type 3 and Type 4. Unlike the results of the previous four types (Type 1–Type 4), the curve of the changes in temperature with time (Figure 7c), shows that a second rise in temperature occurred after the first rise. Moreover, the maximum temperature rise due to impact energy was the lowest in Type 5 compared to the other types. This seems to be an effect of the EVA sheet being laminated both sides on the outside.

**Figure 8:**
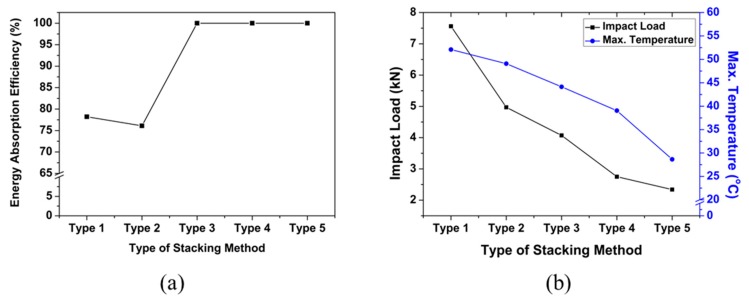
Comparative results for impact test: (**a**) energy absorption efficiency of each type and (**b**) impact load and temperature of each type.

The comparative analysis between the results of the drop test and the temperature generated at the time of breakage are shown in Figure 8. Figure 8a shows the energy absorption efficiency of each lamination type, and Figure 8b shows the impact load and temperature results. These results show that 100% of the impact energy was absorbed in Type 3–5 specimens. In Figure 8b, it can be seen that both the temperature and the impact load dropped as the number of EVA sheets was increased because the heat was dissipated in the process of energy transfer to the specimen during the impact as the energy was transmitted to the impactor without being released.

### 3.2. Compression after Impact Test Results

**Figure 9:**
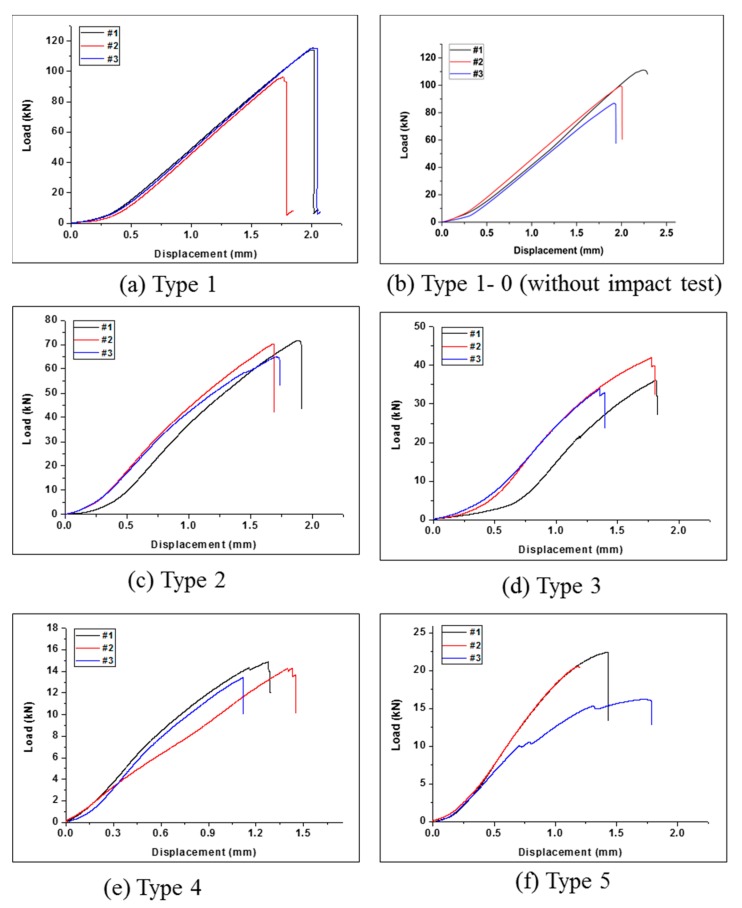
Load and displacement curve of the CAI test, (**a**) Type 1 (24 CFRP), (**b**) Type1-0 (24 CFRP, without impact test), (**c**) Type 2 (20CFRP:2EVA), (**d**) Type 3 (16CFRP:4EVA), (**e**) Type 4 (8CFRP:8EVA, inside), (**f**) Type 5 (8CFRP:8EVA, outside).

**Figure 10:**
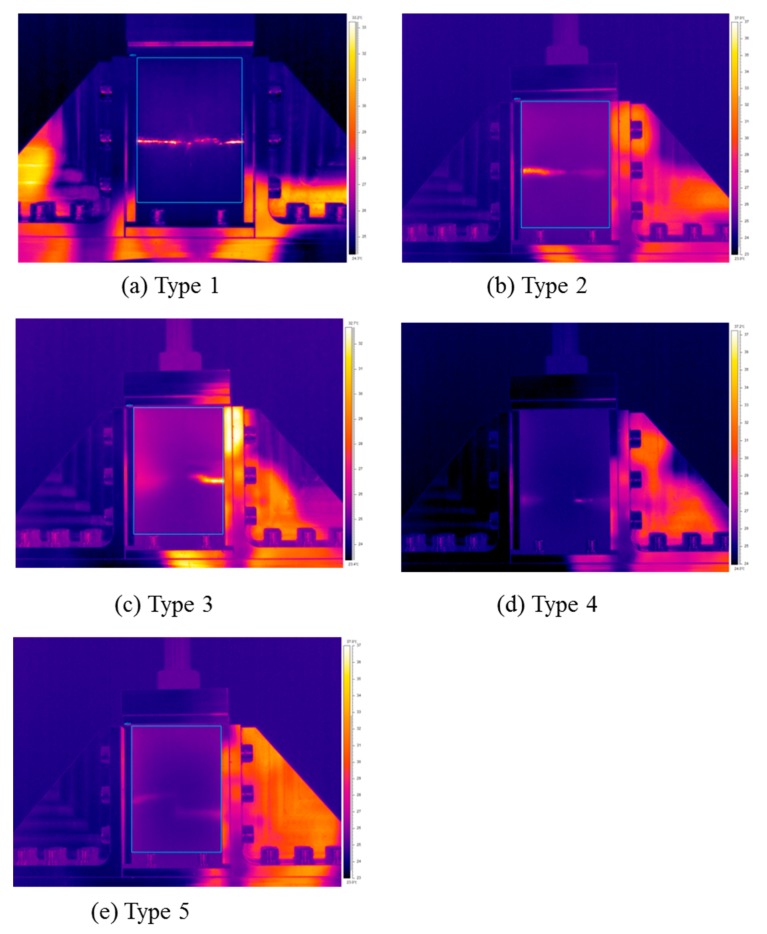
Thermal images of CAI tested specimens, (**a**) Type 1 (24 CFRP), (**b**) Type 2 (20CFRP:2EVA), (**c**) Type 3 (16CFRP:4EVA), (**d**) Type 4 (8CFRP:8EVA, inside), (**e**) Type 5 (8CFRP:8EVA, outside).

Simultaneously with the CAI test, a thermal imaging camera was used to compare and analyze the CAI intensity and the maximum temperature of each type. Figure 9a and Table 10 show the CAI results for Type 1. The average residual compressive strength of Type 1 was 208.31 MPa, and the maximum temperature at the time of breakage was 49.98 °C. For comparison, the sample version of Type 1, referred as Type 1-0 in Figure 9b, was tested under the same condition as CAI test but without prior impact testing. The test results of Type 1-0 are shown in Table 11. The average residual compressive strength of Type 1-0 was 233.67 MPa, which is 10.85% higher than that of Type 1. This implies that the drop impact test reduced the compressive strength of the composites. Figure 10a shows the radiation energy generated during breakage, which was visualized as temperature using a thermal imaging camera. The fracture occurred as the cracks propagated from the drop site to both ends. Note that the temperature change in Type 1-0 during the compressive test could not be detected by the thermographic camera; therefore, the temperature results in Type 1-0 were not reported.

Figure 9c and Table 12 show the residual compression test results of Type 2 with cross-laminated EVA and CFRP. The residual compressive strength was 145.37 MPa, which was ~70% lower than that of pure CFRP, and was 33% lower than that of Type 1. The thermal image shown in Figure 10b indicates that the fracture occurred at the cross-section of the specimens and the fracture tended to be extend horizontally from one side to the other.

Figure 9d and Table 13 show the residual compression test results of Type 3. The compressive strength was found to be 85.97 MPa. It was confirmed that the strength was about 40% lower than that of Type 2 laminated with two sheets of EVA. The thermal image in Figure 10c confirmed that the cracks propagated to both ends from the impact site to the center as in the previous results.

Figure 9e and Table 14 show the residual compression test results of Type 4. The compressive strength was found to be 34.17 MPa. It was confirmed that the strength was about 40% lower than that of Type 3 laminated with four sheets of EVA. The thermal image in Figure 10d confirmed that the cracks propagated to both ends from the impact site in the center, as in the previous results. 

Figure 9f and Table 15 show the residual compression test results of Type 5. The compressive strength was found to be 49.19 MPa. It was confirmed that the strength was about 10% higher than that of Type 4 laminated with the same amount of EVA sheet. Note that, in Type 4, the EVA sheets were laminated inside the composite; however, in Type 5, the EVA sheets were laminated on the outside, resulting in the different lamination structure. The thermal image in Figure 10e confirmed also that the cracks propagated to both ends from the impact site in the center, as in the previous results.

**Figure 11:**
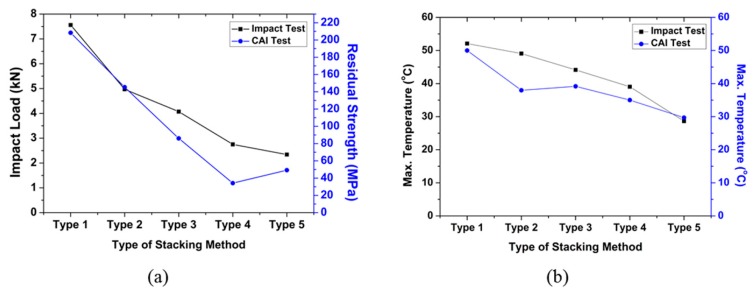
Comparison of the test results: (**a**) compression residual strength and impact load of each type, and (**b**) compression residual strength and temperature of each type.

The results of the drop impact test and the CAI test for each type are shown in the graphs in Figure 11. Figure 11a shows the result of comparison of the impact load applied to the specimen during the drop impact and the residual compressive strength obtained by the CAI test on the specimen after the impact. The results showed that the impact load gradually decreased from Type 1 to Type 5. On the other hand, the residual compressive strength partially decreased from Type 1 to type 4 and then increased in the case of Type 5. This increase of the compressive strength of Type 5 with respect to Type 4 might be due to lamination of EVA sheets on the outer surface of the composite. Note that Type 3–5 had lower impact load and lower residual strength; however, as seen previously, these composites had an impact energy absorption efficiency of 100%. Therefore, it can be judged that the lower load impact load and high impact energy efficiency can be associated with lower residual compressive strength.

Figure 11b shows the maximum temperature obtained by the two tests for each type. The temperature decreased as the amount of EVA sheet increased after impact test and after CAI test. Experimental results of Type 4 and Type 5 specimens in which the EVA sheet was laminated inside and outside, respectively, were examined. The impact strength and maximum temperature were higher in the Type 4 specimen with the EVA sheet laminated inside the composite, whereas the residual compressive strength was higher in the Type 5 specimen. Therefore, it was deduced that the lamination structures of the CFRP and EVA have a significant effect on the mechanical impact behavior of the composites. The results of this study confirmed that the EVA sheet helped alleviate the impact applied to the composite material. In addition, it is expected to be applicable to the design of carbon composite materials for impact mitigation.

**Figure 12:**
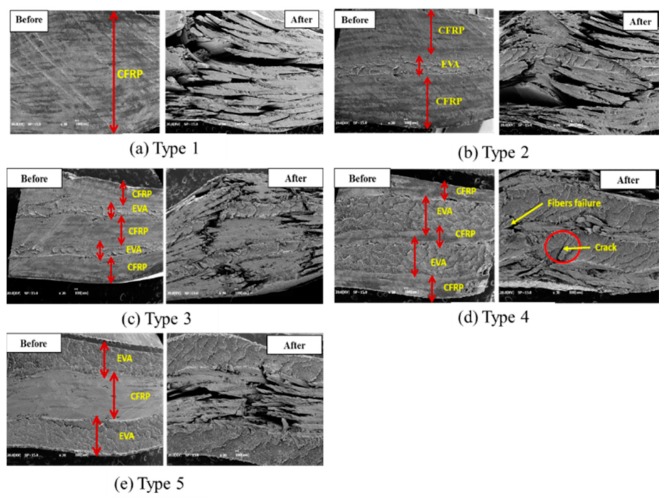
SEM images of each type before and after testing, (**a**) Type 1 (24 CFRP), (**b**) Type 2 (20CFRP:2EVA), (**c**) Type 3 (16CFRP:4EVA), (**d**) Type 4 (8CFRP:8EVA, inside), (**e**) Type 5 (8CFRP:8EVA, outside).

Figure 12 shows the microstructure of the composites before and after testing. The interface of between CFRP stacks and EVA stacks can distinctively be seen on the SEM images before testing. The SEM observation shows that, after testing, severe damages occurred in CFRP stacks as compared to EVA sheets. Those damages were reduced as the number of EVA sheets increased. The failure of CFRP occurred by breakage of carbon fiber due to impact drop and compression, which can be caused by the brittleness of carbon fiber. However, the failure of EVA occurred through the initiation of a crack. This implies that the EVA resists much more on impact and compression testing than the CFRP. Therefore, the EVA sheets improved the resistance to impact load of the composites.

## 4. Conclusions

CFRP is known to exhibit higher strength and stiffness compared to metals; however, due to its brittleness, its application is limited. In this study, a drop impact test was performed by laminating flexible EVA sheets with CFRP prepreg, and a CAI test was performed to confirm the residual compressive strength. At the same time, images were acquired using a thermal imaging camera to analyze the fracture mode and thermal characteristics. The conclusions drawn are as follows:

1. The drop impact test on CFRP laminated with EVA showed that there was no significant difference in the impact absorption efficiencies when two sheets of highly flexible EVA were laminated. However, the Type 3 specimen laminated with four sheets of EVA demonstrated an impact absorption efficiency of 100%. In addition, it was confirmed that there was no significant difference between CFRP laminated with EVA sheets on the outside and inside. The thermal energy generated during the impact was proportional to the impact load. In the thermal images taken during impact, the behavior of the puncture mode was observed as the number of laminated EVA sheets increased.
2. As a result of the CAI test conducted to measure the residual compressive strength of the test specimen after the drop impact test, 50% to 30% of the strength was reduced compared to the original compressive test specimen. A higher number of EVA layers resulted in lower CAI strength values.
3. Thermal images were acquired and analyzed to assess the failure mode and thermal characteristics of the drop impact test and the CAI test. With the CAI specimens, it was difficult to identify an apparent tendency because random damage was already generated due to the drop impact. However, the impact mode and the direction of crack propagation were confirmed by analyzing the images. SEM image observation showed that the CFRP was prone to large fracture damage, whereas the EVA sheets tended to slow the fracture of the composites during the testing.
4. The study results confirmed that the application of EVA sheets, with excellent flexibility, to the carbon composite materials is helpful in mitigating external impact. However, the results may vary depending on the lamination conditions of EVA sheets. By selecting the optimal conditions through prior review, carbon composite materials can be applied for impact mitigation. In this study, it was found that, based on EVA sheets, the composite Type 3 is the optimum structure for high-impact energy absorption efficiency with good compression strength.

## Figures and Tables

**Table 1:** Composition of WSN-3k [6].

Thickness (mm)	Fiber Areal Weight (g/m^2^)	Resin Content (%)	Total Weight (g/m^2^)
0.227	240	41	336

**Table 2:** Mechanical properties of ethylene vinyl acetate (EVA) sheet [6].

**VA Content (%)**	**Weight**	**Tensile Strength (kg/cm^2^)**	**Elongation (%)**
33	0.96	85	800
**Tensile Modulus (kg/cm^2^)**	**Hardness**	**Softening Point (Vicat, °C)**	**Thickness (mm)**
900	60	Below 40	0.45

**Table 3:** Laminated arrangement method. CFRP—carbon-fiber-reinforced plastic.

Type 1	Type 2	Type 3	Type 4	Type 5
24 (CFRP)	20 (CFRP):2 (EVA)	16 (CFRP):4 (EVA)	8 (CFRP):8 (EVA)	8 (CFRP):8 (EVA)
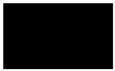	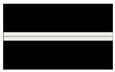	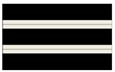	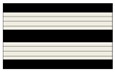	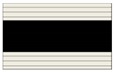

**Table 4:** Experimental conditions for the drop weight impact test.

Impact Velocity (m/s)	Drop Weight (kg)	Impact Energy (J)	Test Height (m)	Room Temperature (°C)
3.92	5.219	39.96	0.79	+23 ± 2

**Table 5:** Impact test results of Type 1.

#	Impact Maximum Load (kN)	Impact Energy at Maximum. Load (J)	Absorbed Energy (J)	Impact Absorption Efficiency (%)	Maximum Temperature (°C)
1	7.14	40.47	31.59	78.06	51.73
2	7.69	40.15	31.95	79.56	57.13
3	7.85	40.22	30.97	77.01	47.40
Average	7.56	40.28	31.50	78.21	52.09
SD	0.30	0.14	0.40	1.05	3.98

**Table 6:** Impact test results of Type 2.

#	Impact Maximum Load (kN)	Impact Energy at Maximum Load (J)	Absorbed Energy (J)	Impact Absorption Efficiency (%)	Maximum Temperature (°C)
1	5.16	40.57	30.21	74.44	52.49
2	4.88	40.97	31.48	76.85	49.91
3	4.89	40.91	31.49	76.98	44.84
Average	4.97	40.82	31.06	76.09	49.08
SD	0.13	0.17	0.60	1.17	3.18

**Table 7:** Impact test results of Type 3.

#	Impact Maximum Load (kN)	Impact Energy at Maximum Load (J)	Absorbed Energy (J)	Impact Absorption Efficiency (%)	Maximum Temperature (°C)
1	3.74	41.05	41.05	100.00	41.75
2	4.61	41.25	41.24	99.99	45.48
3	3.85	40.86	40.86	100.00	45.19
Average	4.07	41.05	41.05	100.00	44.14
SD	0.38	0.16	0.16	0.00	1.70

**Table 8:** Impact test results of Type 4.

#	Impact Maximum Load (kN)	Impact Energy at Maximum Load (J)	Absorbed Energy (J)	Impact Absorption Efficiency (%)	Maximum Temperature (°C)
1	2.85	30.04	30.04	100.00	40.44
2	2.72	30.91	30.91	100.00	38.11
3	2.69	30.01	30.01	100.00	38.60
Average	2.75	30.32	30.32	100.00	39.05
SD	0.07	0.42	0.42	0.00	1.01

**Table 9:** Impact test results of Type 5.

#	Impact Maximum Load (kN)	Impact Energy at Maximum Load (J)	Absorbed Energy (J)	Impact Absorption Efficiency (%)	Maximum Temperature (°C)
1	2.55	27.91	27.91	100.00	28.29
2	2.22	27.85	27.85	100.00	29.21
3	2.26	28.23	28.23	100.00	28.42
Average	2.34	28.00	28.00	100.00	28.64
SD	0.14	0.16	0.16	0.00	0.41

**Table 10:** Compression after impact (CAI) test results of Type 1.

#	Area (mm^2^)	Load (kN)	Compressive Residual Strength (MPa)	Maximum Temperature (°C)
1	520.73	114.24	219.39	58.82
2	520.57	96.36	185.10	42.87
3	524.41	115.60	220.44	48.25
Average	519.93	100.88	208.31	49.98
SD	4.33	18.00	20.10	8.11

**Table 11:** CAI test results of Type 1-0 (without impact test).

#	Area (mm^2^)	Load (kN)	Compressive Residual Strength (MPa)	Maximum Temperature (°C)
1	425.67	111.12	261.05	58.82
2	426.82	99.48	233.07	42.87
3	419.64	86.82	206.89	48.25
Average	424.04	99.14	233.67	49.98
SD	3.86	12.15	27.08	8.11

**Table 12:** CAI test results of Type 2.

#	Area (mm^2^)	Load (kN)	Compressive Residual Strength (MPa)	Maximum Temperature (°C)
1	474.81	71.66	150.92	34.70
3	473.51	70.18	148.21	37.77
3	474.51	65.00	136.98	41.46
Average	474.28	68.95	145.37	37.97
SD	0.68	3.50	7.39	3.39

**Table 13:** CAI test results of Type 3.

#	Area (mm^2^)	Load (kN)	Compressive Residual Strength (MPa)	Maximum Temperature (°C)
1	431.29	36.18	83.89	41.10
2	434.91	41.98	96.53	42.78
3	437.44	33.90	77.50	33.61
Average	434.54	37.35	85.97	39.16
SD	3.09	4.17	9.68	4.88

**Table 14:** CAI results of Type 4.

#	Area (mm^2^)	Load (kN)	Compressive Residual Strength (MPa)	Maximum Temperature (°C)
1	416.75	14.88	35.70	35.03
2	413.04	14.28	34.57	35.93
3	415.75	13.40	32.23	34.13
Average	415.18	14.19	34.17	35.03
SD	1.92	0.74	1.77	0.90

**Table 15:** CAI test results of Type 5.

#	Area (mm^2^)	Load (kN)	Compressive Residual Strength (MPa)	Maximum Temperature (°C)
1	400.20	22.46	56.12	31.52
2	401.88	20.58	51.21	28.17
3	403.68	16.24	40.23	29.34
Average	401.92	19.76	49.19	29.70
SD	1.74	3.19	8.14	1.73
